# Bace1 Deletion in the Adult Reverses Epileptiform Activity and Sleep–wake Disturbances in AD Mice

**DOI:** 10.1523/JNEUROSCI.2124-22.2023

**Published:** 2023-08-30

**Authors:** Annie Y. Yao, Patrick J. Halloran, Yingying Ge, Neeraj Singh, John Zhou, James Galske, Wanxia He, Riqiang Yan, Xiangyou Hu

**Affiliations:** Department of Neuroscience, University of Connecticut Health Center, Farmington, Connecticut 06030

**Keywords:** Alzheimer’s disease, BACE1, NREM, epileptic, REM, sleep disturbance

## Abstract

Alzheimer’s disease (AD) increases the risk for seizures and sleep disorders. We show here that germline deletion of β-site amyloid precursor protein (APP) cleaving enzyme-1 (BACE1) in neurons, but not in astrocytes, increased epileptiform activity. However, *Bace1* deletion at adult ages did not alter the normal EEG waveform, indicating less concern for BACE1 inhibition in patients. Moreover, we showed that deletion of *Bace1* in the adult was able to reverse epileptiform activity in 5xFAD mice. Intriguingly, treating 5xFAD and APP^NL-G-F/NL-G-F^ (APP KI) mice of either sex with one BACE1 inhibitor Lanabecestat (AZD3293) dramatically increased epileptiform spiking, likely resulting from an off-target effect. We also monitored sleep–wake pathologies in these mice and showed increased wakefulness, decreased non-rapid eye movement sleep, and rapid eye movement sleep in both 5xFAD and APP KI mice; BACE1 inhibition in the adult 5xFAD mice reversed plaque load and sleep disturbances, but this was not seen in APP KI mice. Further studies with and without BACE1 inhibitor treatment showed different levels of plaque-associated microgliosis and activated microglial proteins in 5xFAD mice compared with APP KI mice. Together, BACE1 inhibition should be developed to avoid off-target effect for achieving benefits in reducing epileptic activity and sleep disturbance in Alzheimer’s patients.

**SIGNIFICANCE STATEMENT** BACE1 is widely recognized as a therapeutic target for treating Alzheimer’s disease patients. However, BACE1 inhibitors failed in clinical trials because of inability to show cognitive improvement in patients. Here we show that BACE1 inhibition actually reduces sleep disturbances and epileptic seizures; both are seen in AD patients. We further showed that one of clinically tested BACE1 inhibitors does have off-target effects, and development of safer BACE1 inhibitors will be beneficial to AD patients. Results from this study will provide useful guidance for additional drug development.

## Introduction

Alzheimer’s disease (AD), the most common cause of age-related dementia, is a debilitating neurodegenerative disease that leads to progressive memory loss, cognitive impairment, and ultimately death (Golde, 2022). AD patients have increased risk for seizures and neuronal network abnormalities beyond the risk associated with normal aging (Palop and Mucke, 2009; Scarmeas et al., 2009). It has been shown that 10%-22% of AD patients experience at least one unprovoked seizure during their disease courses (Mendez and Lim, 2003). Late-onset sporadic AD is associated with a 6- to 10-fold increased risk of developing generalized- and partial-onset unprovoked seizures compared with the general population (Hesdorffer et al., 1996). In early-onset dominant familial AD, seizures occur even more frequently, with one prospective study showing a striking 87-fold increase in seizures (Amatniek et al., 2006). Seizures in AD exhibit an exceptionally high recurrence risk and are associated with a poor clinical symptom course (Vöglein et al., 2020). Since these neuronal network abnormalities may underlie and/or exacerbate cognitive deficits in AD, seizures are thus an important target for clinical intervention.

The abnormal accumulation of cerebral amyloid-β (Aβ) is viewed as an early event leading to AD (Jack et al., 2013). BACE1 is the enzyme responsible for initiating the production of Aβ, and inhibition of BACE1 can reduce Aβ generation. However, BACE1 is also an indispensable protein for normal physiological functions in the brain, including the control of neurogenesis, astrogenesis, myelination, and synaptic function (Hampel et al., 2021). Global germline *Bace1*-null mice exhibit spontaneous epilepsy and epileptiform spike-wave discharges (Hitt et al., 2010; Hu et al., 2010). Since BACE1 is widely expressed in the brain across many cell types, including neurons, oligodendrocytes, microglia, and astrocytes (Singh et al., 2022b), it is still unknown whether *Bace1* deletion specifically in neurons or astrocytes contributes to the development of seizures. Additionally, while previous studies have shown that germline *Bace1* deletion in mice leads to seizures and abnormal epileptiform discharges, it is unknown whether this phenotype will arise when *Bace1* is globally deleted starting at adult ages. Most importantly, it is unclear whether *Bace1* deletion starting at adult ages can ameliorate the seizure phenotype in AD mouse models.

In addition to seizures, sleep disorders are also frequently observed at various stages of AD progression (Varga et al., 2016; De Gennaro et al., 2017; Minakawa et al., 2019; Mander, 2020). More than 60% of AD patients exhibit sleep disturbances (Guarnieri et al., 2012), with AD patients’ sleep changes greatly exceeding the changes seen in age-matched elderly controls (Prinz et al., 1982; Petit et al., 2004). Symptoms reported by caregivers and patients, and corroborated by polysomnographic studies, include sleep fragmentation, increased nocturnal awakenings, sleeping either longer or shorter than normal, low sleep efficiency, insomnia, and late sleep onset (Ju et al., 2014). This is accompanied by sleep architecture changes as indicated by EEG alteration, including decreased duration of rapid eye movement (REM) sleep and diminished slow-wave sleep (Loewenstein et al., 1982; Prinz et al., 1982; Bliwise et al., 1989; Petit et al., 2004). Importantly, sleep disturbances predict the risk of AD, presenting during the asymptomatic or preclinical stages (Ju et al., 2014; Minakawa et al., 2019; Mander, 2020), and drive disease pathology (Musiek et al., 2015, 2018).

Here, we showed that germline *Bace1* deletion in neurons, but not in astrocytes, increased epileptiform activity, while *Bace1* deletion at adult ages minimally altered the normal EEG waveform. Importantly, deleting *Bace1* in the adult was able to reverse epileptiform activity in 5xFAD mice. Intriguingly, treatment with one of the brain-penetrable BACE1 inhibitors, Lanabecestat (AZD3293), dramatically increased epileptiform spiking in both 5xFAD and APP^NL-G-F/NL-G-F^ (APP KI) mice, likely related to an off-target effect from the compound. Finally, we characterized differences in sleep–wake disturbances and microglial activation in 5xFAD and APP KI mice, and evaluated how the BACE1 inhibitor treatment differentially attenuated plaque load and sleep disturbances in the two AD mouse models.

## Materials and Methods

### Experimental animals

For the seizure studies focused on BACE1 in different cell populations, conditional *Bace1* KO (Bace1^fl/fl^) mice were crossed with three Cre lines to target neuronal, astrocyte, or global adult Bace1 deletion. To target *Bace1* deletion to postnatal neuronal populations, we crossed BACE1^fl/fl^ mice with heterozygous Thy1-cre (RRID:IMSR_JAX:006143) mice to generate Bace1^fl/fl/thy1Cre^ heterozygous mice. To target astrocytic *Bace1* deletion, we crossed Bace1^fl/fl^ mice with heterozygous Aldh1-cre/ER^T2^ BAC transgenic (RRID:IMSR_JAX:029655) mice to generate Bace1^fl/fl/Aldh1CreERT2^ mice, which express tamoxifen-inducible Cre recombinase to delete *Bace1* in astrocytes. For adult whole-body inducible *Bace1* deletion, we crossed Bace1^fl/fl^ mice with UBC-Cre/*ER^T2^* (RRID:IMSR_JAX:007001) mice to generate Bace1^fl/fl/UbcCreER^ mice, for tamoxifen-inducible, ubiquitin C promoter-driven Cre-ERT2 expression in broad cell populations. Previously, we found that UBC-Cre/*ER^T2^* mice have a Cre recombinase leakage issue (Hu et al., 2018). BACE1 reduction starts at 1 month of age and is reduced by ∼50% at 2 months and ∼80% at 4 months in Bace1^fl/fl/UbcCreER^ mice. For these cohorts, all transgenic animals were compared with littermate Bace1^fl/fl^ controls.

For the seizure and sleep studies in two different AD mouse lines, we used 5xFAD mice and APP KI mice. 5xFAD (RRID:MMRRC_034840-JAX) mice express human APP and PSEN1 transgenes with five AD-linked mutations: the Swedish (K670N/M671L), Florida (I716V), and London (V717I) mutations in APP, and the M146L and L286V mutations in PSEN1. To conditionally delete *Bace1* in a 5xFAD background, we crossed Bace1^fl/fl/5xFAD^ mice with Bace1^fl/fl/UbcCreER^ mice to generate Bace1^fl/fl/UbcCreER/5xFAD^ mice. APP KI mice express an APP construct with a humanized Aβ region that includes the Swedish (KM670/671NL), Iberian (I716F), and Arctic (E693G) mutations. Heterozygous APP^NL-G-F/wt^ mice were crossed to generate homozygous APP^NL-G-F/NL-G-F^ mice and APP^wt/wt^ control mice.

Mice were maintained on a C57/Bl6J background and housed on a 12 h light/12 h dark cycle with access to food and water *ad libitum*. Mice of both sexes were used. Unless otherwise indicated, there was no significance between genders, and data presented are the means of both male and female animals. All animal use and procedures were performed according to the Institutional Animal Care and Use protocols at UConn Health Center, Farmington, and in compliance with the guidelines established by the *Guide for the care and use of laboratory animals*, as adopted by the National Institutes of Health. Experimenters were blinded to genotypes or treatment conditions for data collection and analysis.

### Animal surgery and EEG

Three-month-old Bace1^fl/fl/Aldh1CreERT2^ mice were intraperitoneally injected with tamoxifen (Sigma, T5648, 20 mg/ml dissolved in peanut oil) at a dose of 75 mg/kg body weight for 5 consecutive days. At 4 months of age, Bacce1^fl/fl/Aldh1CreERT2^, Bace1^fl/fl/thy1Cre^, Bace1^fl/fl/UbcCreER^, and control Bace1^fl/fl^ mice underwent EEG/EMG implantation surgery, as described previously (Hu et al., 2010). Briefly, mice were anesthetized via isoflurane pump (RWD, Gas Evacuation Apparatus R546W) and held in a stereotaxic frame fitted with a mouse adaptor (Stoelting). The skull was exposed, and the EEG head mount was fixed with four screws, all of which was secured with dental ceramic compound. Two EMG wire electrodes were also inserted contralaterally into the nuchal musculature. After surgery, the mouse was allowed to recover for 1 week before EEG/EMG recording. Video-EEG/EMG recordings with synchronized video were performed using a preamplifier connected with the animal’s head mount and a commutator, which was attached to the Data Acquisition and Control System (Pinnacle Technology). All mice were maintained on a 12 h light/12 h dark cycle and received 2 d of continuous recording. Amplified EEG and EMG signals were digitally collected, processed, and visualized with Sirenia Acquisition software (Pinnacle Technology). Epileptiform activity was manually scored with Sirenia Seizure Pro.

### BACE1 inhibitor AZD3293 treatment and EEG

Nine-month-old 5xFAD mice and APP KI mice similarly underwent EEG/EMG implantation surgery as described above. For cohorts of 5xFAD mice and APP KI mice treated with the BACE1 inhibitor AZD3293, drug treatment was performed by 1 mg/kg oral gavage once per day for 60 consecutive days (starting at 7 months of age) before EEG/EMG implantation surgery. After 1 week of recovery after surgery, video-EEG/EMG recordings were performed in 12 h light/12 h dark cycles for 2 d.

### Sleep–wake scoring and analysis

Sleep stage and wake analyses were performed on 24 h of the EEG recordings. EEG traces were scored automatically and confirmed manually for WAKE, non-REM (NREM), and REM by an investigator blinded to genotype using a 10 s epoch duration and Sirenia Sleep Pro software (Pinnacle Technology). Data were first analyzed using cluster scoring, evaluating power by specific frequency bands (e.g., δ, theta, α, β, and γ) for the EEG and EMG channels to identify bouts of sleep, wake, and transition periods. The scoring of each 10 s epoch was then confirmed through visual inspection by evaluating the recording and corresponding spectral plot. WAKE was defined by low-amplitude EEG, dominant EEG frequency >4 Hz, with mixed high-frequency components and high-amplitude EMG. NREM sleep was defined by high-amplitude EEG, dominant EEG frequency <4 Hz, with predominant δ and theta components, and low-amplitude EMG. REM was defined by low-amplitude EEG, a dominant theta frequency (4-8 Hz), uniform EEG waveforms, low-amplitude EMG indicating muscle atonia with occasional muscle twitches, and occurring at a transition from NREM to wake. An epoch was defined according to which state was >50% of the 10 s epoch. Percentage of time spent in each sleep or wake state was calculated by dividing by total time (24 h). Statistical analyses were performed using GraphPad Prism 7.0a (GraphPad Software). Statistical significance was set at *p* ≤ 0.05. Statistical significance was determined by one-way ANOVA and followed by Tukey’s multiple comparisons test. All data are expressed as mean ± SEM.

### Extracellular field potential recording with 4-AP-induced ictogenesis assay

Brain slices were prepared following previously described protocols (Hu et al., 2010; Panuccio et al., 2018). Briefly, horizontal hippocampal slices (300 μm thickness) were prepared from the brains of P40-P45 WT and *Bace1*-null mice in ice-cold, oxygenated 95% O_2_/5% CO_2_ aCSF consisting of the following (in mm): 115 NaCl, 2 KCl, 1.25 KH_2_PO_4_, 1.0 MgSO_4_, 2.0 CaCl_2_, 26 NaHCO_3_, 10 glucose, and 1.0 L-ascorbic acid. After a 1 h recovery period in regular aCSF, the brain slice was transferred to another holding chamber containing aCSF with 100 μm 4-AP to incubate for another hour. For the recordings, the brain slice was placed onto the center of a MED-P515A probe (AutoMate Scientific) with 64 embedded recording sites and perfused with aCSF, and held in place with a harp. Extracellular field potentials were recorded using the MED64 amplifier and multichannel recording system (AutoMate Scientific). Data were collected from the hippocampal CA3 region and then analyzed with MED64 Mobius and Offline Single-Channel Burst Analysis software to quantify spike events (interval to start of burst, burst duration, interval between bursts). A burst was defined as: minimum 15 spikes, minimum 50 ms duration, minimum 100 ms interval between bursts. Statistical analyses were performed using GraphPad Prism (GraphPad Software). Statistical significance was determined by one-way ANOVA and followed by Tukey’s multiple comparisons test. Data are presented as mean ± SEM.

### Immunohistochemistry

Animals were killed at the end of each EEG experiment, and brain tissue was collected as described previously (Singh et al., 2022a). Brains were surgically removed and cut mid-sagittally into equal halves. One half of the brain was fixed in 4% PFA for 24 h and then immersed in 20% sucrose overnight at 4°C. The other half would be used for Western analysis. Brains were then sectioned sagittally (14-16 μm thick) on a cryostat microtome (Fisher Scientific HM525 NX). Sections on slides were washed in PBS 3× for 5 min to remove OCT and then permeabilized with 0.3% Triton X-100 for 30 min, followed by washing with PBS (3× for 5 min). Antigen retrieval was performed by microwaving the sections in 0.05 m citrate-buffered saline, pH 6.0, for 2 min. The sections were blocked with 5% NGS and incubated with the following primary antibodies at a 1:1000 dilution: β-amyloid 1-16, 6E10 (AB_2564652, BioLegend) and ionized calcium-binding adapter molecule 1 (IBA-1, AB_839504, Wako). After washing with PBS (4× for 5 min), sections were incubated with AlexaFluor-conjugated secondary antibody (1:500 in blocking buffer) at room temperature for 2 h. Slides were washed three times in PBS and mounted on a coverslip with Antifade mounting medium.

For the DAB staining, after the primary antibody, sections were incubated with universal biotinylated anti-mouse/rabbit IgG (1:200, Vector Laboratories) at room temperature for 2 h. After washing with PBS (3× for 5 min), sections were incubated with avidin-biotin peroxidase complex (1:200, Vector Laboratories) at room temperature for 1 h. Sections were then incubated with 0.05% DAB (Sigma) with 0.01% H_2_O_2_ in PBS for 5 min. Slides were washed 3 times in PBS and mounted on a coverslip with 60% glycerol.

### Quantification of amyloid plaque load and microglial morphologies

Serial sagittal sections, selected at 10 section intervals starting from the beginning of the hippocampus, were probed with Aβ monoclonal antibody 6E10 and stained with DAB. Images were captured with a Keyence fluorescence microscope. Plaque counting in the cortex and hippocampus was conducted using ImageJ software (National Institutes of Health).

The *z*-stack confocal images (40×) of microglia and amyloid plaques were captured with a Zeiss LSM 800 confocal microscope. Microglia morphologies were quantified using ImageJ software plugin AnalyzeSkeleton. The outputs of this plugin summarized cell morphology in terms of process length and endpoints. Six mice were used for each cohort, and a total of 10 brain sections were analyzed per mouse. Data are presented as mean ± SEM.

### Western blotting

Protein extraction was performed according to previously described procedures (Hu et al., 2007). Brain samples were homogenized in RIPA buffer (50 mm Tris-HCl, pH 7.4, 1% NP-40, 0.25% sodium deoxycholate, 150 mm NaCl, 1 mm EDTA, 1 mm NaF, 1 mm Na_3_VO_4_, and a protease inhibitor cocktail [Roche]) and centrifuged at 13,200 rpm for 90 min. Protein concentrations were determined using a bicinchoninic acid assay kit. Equal amounts of protein were loaded and resolved on 4%-12% SDS–polyacrylamide gels (NuPAGE system, Invitrogen). Subsequently, blots were transferred to nitrocellulose membranes at 100 V for 2 h. The membranes were blocked with 5% BSA for 1 h at room temperature. The membranes were probed with the following primary antibodies at the noted dilutions: 1:1000 APP-C (AB_258409, Sigma); 1:1000 BACE1; 1:50,000 actin (AB_476744, Sigma); 1:1000 CD68 (AB_125212, Abcam); 1:3000 Calnexin (AB_476845, Sigma); 1:500 Apo-E (AB_2892618, Santa Cruz Biotechnology); 1:1000 TREM2 (AB_2799888, Cell Signaling); and 1:1000 IBA-1 (AB_2820254, Cell Signaling). After 24 h primary incubation at 4°C, blots were washed extensively and incubated with HRP-conjugated secondary antibodies and visualized using enhanced chemiluminescence (Fisher Scientific). The antibody-bound protein blots were detected by an iBright 1500 imaging system (Invitrogen). For quantification purposes, band intensities of immunoblots were analyzed using ImageJ software.

### Availability of supporting data

All original data presented in the paper will be made available for review when needed. Research materials will be also made available when required.

## Results

### Neuronal, but not astrocytic, *Bace1* deletion increases epileptiform spikes

**Figure 1.**
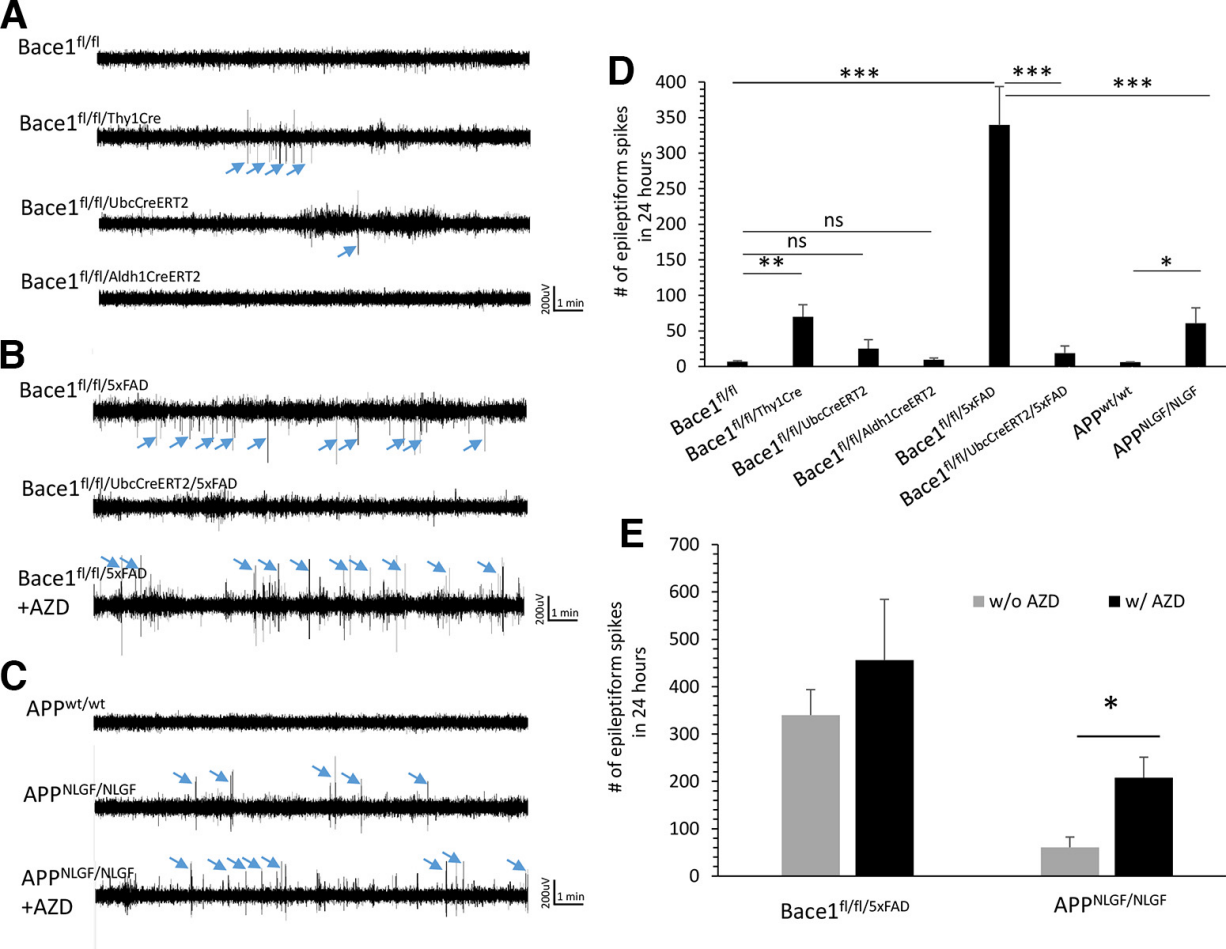
Epileptiform activity was measured in Bace1 neuronal, astrocyte, and adult whole-body Bace1 KO mice, and AD mice with *Bace1* deletion or BACE1 inhibition. ***A***, Representative EEG traces over 15 min from 4-month-old Bace1^fl/fl^, Bace1^fl/fl/Thy1Cre^, Bace1^fl/fl/UbcCreER^, and Bace1^fl/fl/Aldh1l1CreERT2^ mice. Bace1^fl/fl^ mice (*n = *6) exhibited a low-amplitude baseline EEG without spikes, while Bace1^fl/fl/Thy1Cre^ mice exhibited epileptiform spiking (*n = *6). Compared with Bace1^fl/fl/Thy1Cre^ mice, spiking activity was reduced in Bace1^fl/fl/UbcCreER^ mice (*n = *6), and undetectable in Bace1^fl/fl/Aldh1l1CreERT2^ mice (*n = *6). ***B***, ***C***, Representative traces over 15 min from 9-month-old 5xFAD or APP KI (APP^NLGF/NLGF^) mice, with *Bace1* deleted (Bace1^fl/fl/UbcCreER/5xFAD^) or inhibited with AZD3293. Epileptiform spikes were visibly increased in Bace1^fl/fl/5xFAD^ mice, and almost undetectable in *Bace1*-deleted Bace1^fl/fl/UbcCreER/5xFAD^ mice. Epileptiform spikes were also detected in APP KI mice, but less frequent compared with age-matched 5xFAD mice. Treatment with BACE1 inhibitor AZD3293 significantly increased the spiking in both 5xFAD and APP KI mice compared with age-matched counterparts without AZD3293 treatment. Epileptiform spikes are defined as sharp (<50 ms) positive or negative deflections with amplitudes exceeding at least twice the baseline, which are indicated by the blue arrows. ***D***, Comparison of the number of epileptiform spikes in 24 h of recording of the noted genotypes. ***E***, Comparison of the number of epileptiform spikes in 24 h of recording of 9-month-old Bace1^fl/fl/5xFAD^ and APP KI mice, with and without AZD3293 treatment. *p < 0.05; **p < 0.01; ***p < 0.001; one-way ANOVA followed by Tukey’s multiple comparisons test. Data are mean ± SEM.

It has been previously shown that germline *Bace1* deletion in mice leads to seizures and abnormal epileptiform discharges (Hitt et al., 2010; Hu et al., 2010). To determine whether the origin of this epileptiform phenotype is from *Bace1* deletion in neuronal versus astrocytic populations, we performed video-EEG/EMG recordings of 4-month-old freely moving Bace1^fl/fl/Thy1Cre^ and TAM-treated Bace1^fl/fl/Aldh1CreERT2^ mice during a 48 h period. In addition, to assess whether epileptiform activity was seen when *Bace1* was deleted in the adult, we also performed video-EEG/EMG recordings on 4-month-old Bace1^fl/fl/UbcCreER^ mice, which were described previously (Hu et al., 2018). Epileptiform spikes were defined as sharp (<50 ms) positive or negative deflections with amplitudes twice greater than the baseline (see Fig. 1*A–C*, arrows). Representative traces are shown in Figure 1*A–C*, where Bace1^fl/fl^ control mice exhibited a low-amplitude baseline EEG without spiking (Fig. 1*A*, trace 1). When *Bace1* was deleted in Bace1^fl/fl/Thy1Cre^ mouse neuronal populations, epileptiform spikes were visibly increased (Fig. 1*A*, trace 2). Bace1^fl/fl/UbcCreER^ mice, which have progressive deletion of *Bace1* after postnatal day 30, did not exhibit significantly increased spiking compared with control Bace1^fl/fl^ mice (Fig. 1*A*, trace 3). In Bace1^fl/fl/Aldh1CreERT2^ mice, which have *Bace1* conditionally knocked out in astrocytes, epileptic spiking was undetectable (TAM treatment for 1 month beginning at the age of 3 months old; Fig. 1*A*, trace 4).

We went on to quantify epileptiform activity during 24 h of recording, and overall, Bace1^fl/fl/Thy1Cre^ mice presented significantly increased epileptiform spikes compared with age-matched Bace1^fl/fl^ controls (Fig. 1*D*; 69.96 ± 17.03 in Bace1^fl/fl/Thy1Cre^ mice vs 6.88 ± 1.28 in Bace1^fl/fl^ mice, *n *=* *6 pairs, *p* < 0.01). In Bace1^fl/fl/UbcCreER^ mice, where *Bace1* was deleted by ∼80% at this age (Hu et al., 2018), the average spike number was not significantly altered from Bace1^fl/fl^ control (25.14 ± 12.63, *n =*6, *p* = 0.12). Spike number from Bace1^fl/fl/Aldh1CreERT2^ was also comparable to Bace1^fl/fl^ mice (9.41 ± 2.39, *n = *6, *p* = 0.34) (Fig. 1*D*). These results reveal that seizures and abnormal EEGs observed in *Bace1*-null mice can be mainly attributable to germline *Bace1* deletion in neurons, since recombinase activity can be detected in Thy1-Cre mice as early as embryonic day 11 in mice (Campsall et al., 2002). Global deletion of *Bace1* in the adult has significantly reduced seizure phenotype compared with germline-deleted *Bace1*-null mice. Intriguingly, adult-age tamoxifen-induced deletion of *Bace1* in astrocytes appears not to result in the development of seizure activity.

### Increased epileptiform spiking in AD mice is reversed by *Bace1* deletion

Spontaneous seizures and epileptiform spike discharges are observed in rodent models of AD, but it is unclear how BACE1 inhibitors would alter these observed EEG abnormalities. To answer this question, we used video-EEG/EMG recordings of two AD mouse models: (1) conditional deletion of *Bace1* in 5xFAD mice in the adult stage and comparing Bace1^fl/fl/5xFAD^ with Bace1^fl/fl/UbcCreER/5xFAD^ mice; and (2) 5xFAD and APP KI mice treated with and without the BACE1 inhibitor AZD3293 (Fig. 1*B–E*).

Bace1^fl/fl/5xFAD^ mice showed ictal-like discharges, such as spikes, polyspikes, and spike-waves (Fig. 1*B*). Epileptiform spikes were significantly increased in Bace1^fl/fl/5xFAD^ mice compared with age-matched Bace1^fl/fl^ littermates (339.80 ± 53.85 spikes in Bace1^fl/fl/5xFAD^ mice vs 6.88 ± 1.28 in Bace1^fl/fl^ mice, *n *=* *6, *p* < 0.001; Fig. 1*D*). In adult-age *Bace1* deleted 5xFAD mice (Bace1^fl/fl/5xFAD/UbcCreER^), the EEG spiking activity was significantly reduced by 95.5% compared with 5xFAD (18.53 ± 10.11 spikes in Bace1^fl/fl/5xFAD/UbcCreER^, *n = *6), essentially returning back to Bace1^fl/fl^ control levels (Fig. 1*B*,*D*). This indicates that deleting *Bace1* in the adult can reverse seizure activity in an AD model.

Furthermore, we found that APP KI mice exhibited 60.72 ± 21.68 spikes over 24 h, which was significantly increased compared with age-matched APP^wt/wt^ controls (Fig. 1*C*,*D*, *n* = 6, *p* < 0.05). It should be noted that APP KI mice showed less spiking than 5xFAD mice (Fig. 1*D*, *p* < 0.001). Our findings of epileptiform seizures in these two AD models is consistent with prior studies of seizures observed in AD mouse models (Moechars et al., 1996; Lalonde et al., 2005; Siwek et al., 2015; Reyes-Marin and Nuñez, 2017). The reason of different epileptiform spiking levels between these two AD mouse models remains to be understood, but overexpression of APP transgene in 5xFAD mice is likely one of reasons (Born et al., 2014).

Since adult-age *Bace1* deletion in 5xFAD mice reduced epileptiform spiking, we might expect that BACE1 inhibitor treatment would reduce spiking in AD mice as well. Surprisingly, we found that treatment with the BACE1 inhibitor AZD3293 increased the spiking in these two different AD mouse models: in Bace1^fl/fl/5xFAD^ mice (456.03 ± 128.64 spikes, *n = *6, *p* = 0.42) and in APP KI mice (207.94 ± 43.07 spikes, *n = *6, *p* < 0.05), compared with age-matched Bace1^fl/fl/5xFAD^ and APP KI mice without AZD3293 treatment, respectively (Fig. 1*E*).

### BACE1 inhibitor AZD3293 increases burst frequency in *Bace1*-null mice in a 4-AP model of ictogenesis

**Figure 2.**
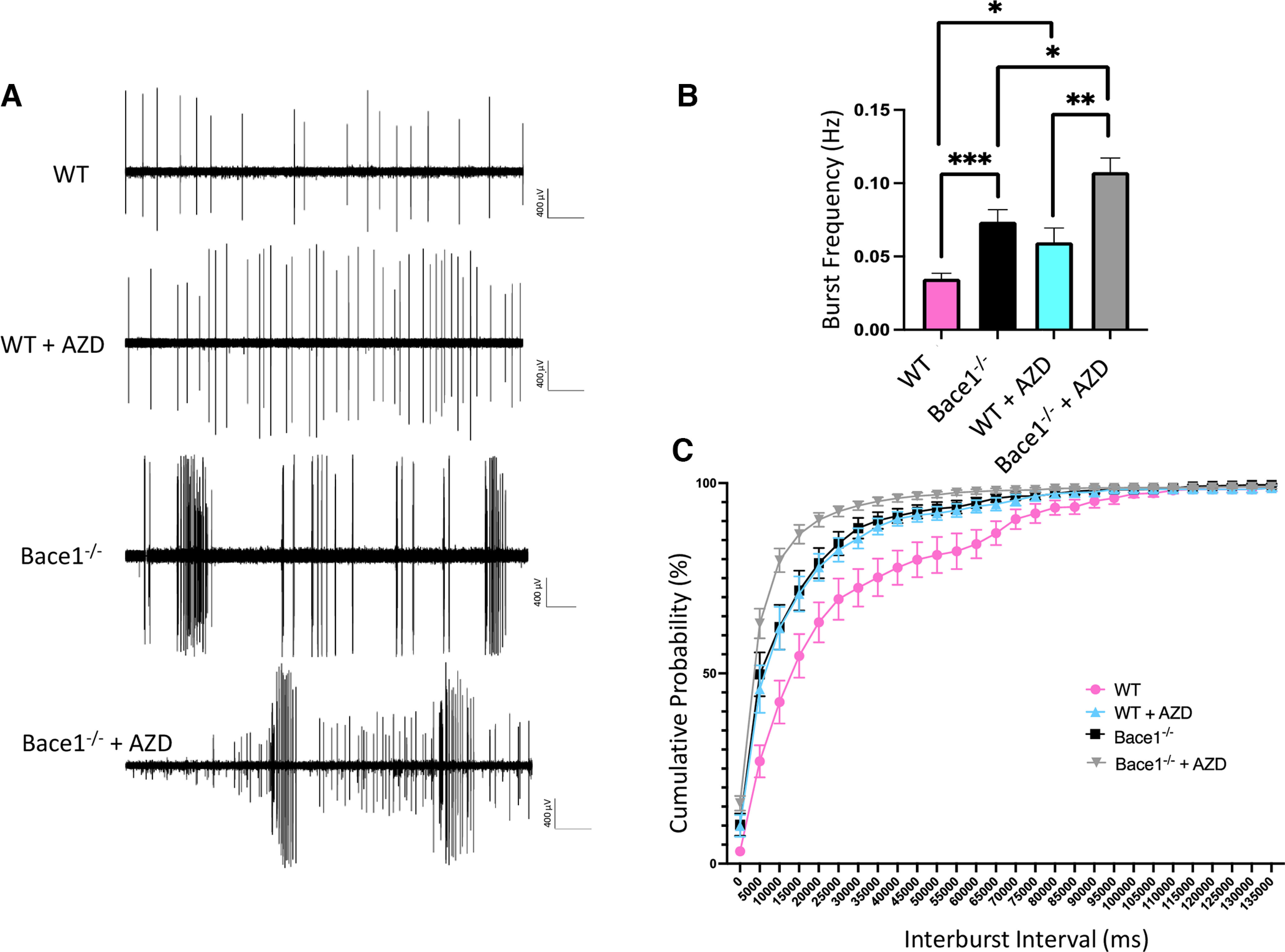
BACE1 inhibitor AZD3293 increased synchronous firing in *Bace1*-null mice. ***A***, Representative traces of 4-AP-induced synchronous firing in the hippocampal CA3 region, recorded in acute brain slices from P40-P45 *Bace1*-null (Bace1^−/−^) mice or WT littermate mice, with or without AZD3293 treatment (*n *=* *6 for each group). Calibration: 400 µV, 60 s. ***B***, Summary histograms quantifying the effect of BACE1 inhibitor AZD3293 on the frequency of bursts (in Hz) of ictal-like events in the hippocampal CA3 area of brain slices from *Bace1*-null mice without (black) or with (gray) AZD3293, and WT without (pink) or with (blue) AZD3293 treatment. In a given recording period, *Bace1*-null mice with AZD3293 treatment displayed the highest frequency of bursts compared with all other groups. ***C***, Cumulative probability of interevent intervals of ictal-like bursts in slices from *Bace1*-null mice without (black) or with (gray) AZD3293, and WT without (pink) or with (blue) AZD3293 treatment. Data collected from 3 animals per treatment, 6-8 slices per animal. *p < 0.05; **p < 0.01; ***p < 0.001; one-way ANOVA followed by Tukey’s multiple comparisons test. Data are mean ± SEM.

Since adult *Bace1* deletion in 5xFAD mice reduced epileptiform spiking, while AZD3293 treatment in adult AD mice increased spiking, we proceeded to investigate whether AZD3293 has an off-target effect by increasing neuronal excitability and enhanced synchronous firing. For example, AZD3293 inhibits BACE2 near equal potency (Eketjäll et al., 2016). To address a possible off-target question, we performed *ex vivo* extracellular field recordings in acute mouse brain slices containing the hippocampus, in which we induced epileptiform activity with the potassium channel blocker 4-AP (100 μm). Using WT and *Bace1*-null mice with or without AZD3293 treatment (1 mg/kg oral gavage once per day over 3 weeks), we detected synchronous epileptiform-like activity throughout the hippocampus after bath application of 4-AP and quantified burst spiking in the CA3 hippocampus (Fig. 2). The frequency of burst firing was increased by more than 2 times in *Bace1*-null mice compared with age-matched WT controls (*p* < 0.001), consistent with our previous observation (Hu et al., 2010). Notably, as illustrated in Figure 2*B*, AZD3293 treatment significantly increased synchronous burst firing frequency in *Bace1*-null mice (0.0737 ± 0.0082 Hz in *Bace1*-null mice vs 0.1075 ± 0.0097 Hz in *Bace1*-null mice + AZD3293, *p* < 0.05). AZD3293 treatment also increased burst firing in WT mice (0.0348 ± 0.0036 Hz in WT mice vs 0.0597 ± 0.0097 Hz in WT mice with AZD3293, *p* < 0.05). The cumulative probability plots of interburst intervals for these mice (Fig. 2*C*) showed that the distribution of bursts in *Bace1*-null mice treated with AZD3293 was left-shifted relative to untreated *Bace1*-null mice, indicating that AZD3293 treatment in these mice led to more frequent burst events.

Together, these data showed that AZD3293 enhances synchronous neuronal firing beyond what is seen in global *Bace1* inhibition. This suggests that chronic treatment of mice with AZD3293 will influence targets other than BACE1. We speculate that this additional effect is related to certain molecular entity in AZD3293, and not necessarily applicable to all BACE1 inhibitors.

### Sleep disturbances in 5xFAD and APP KI mice

**Figure 3.**
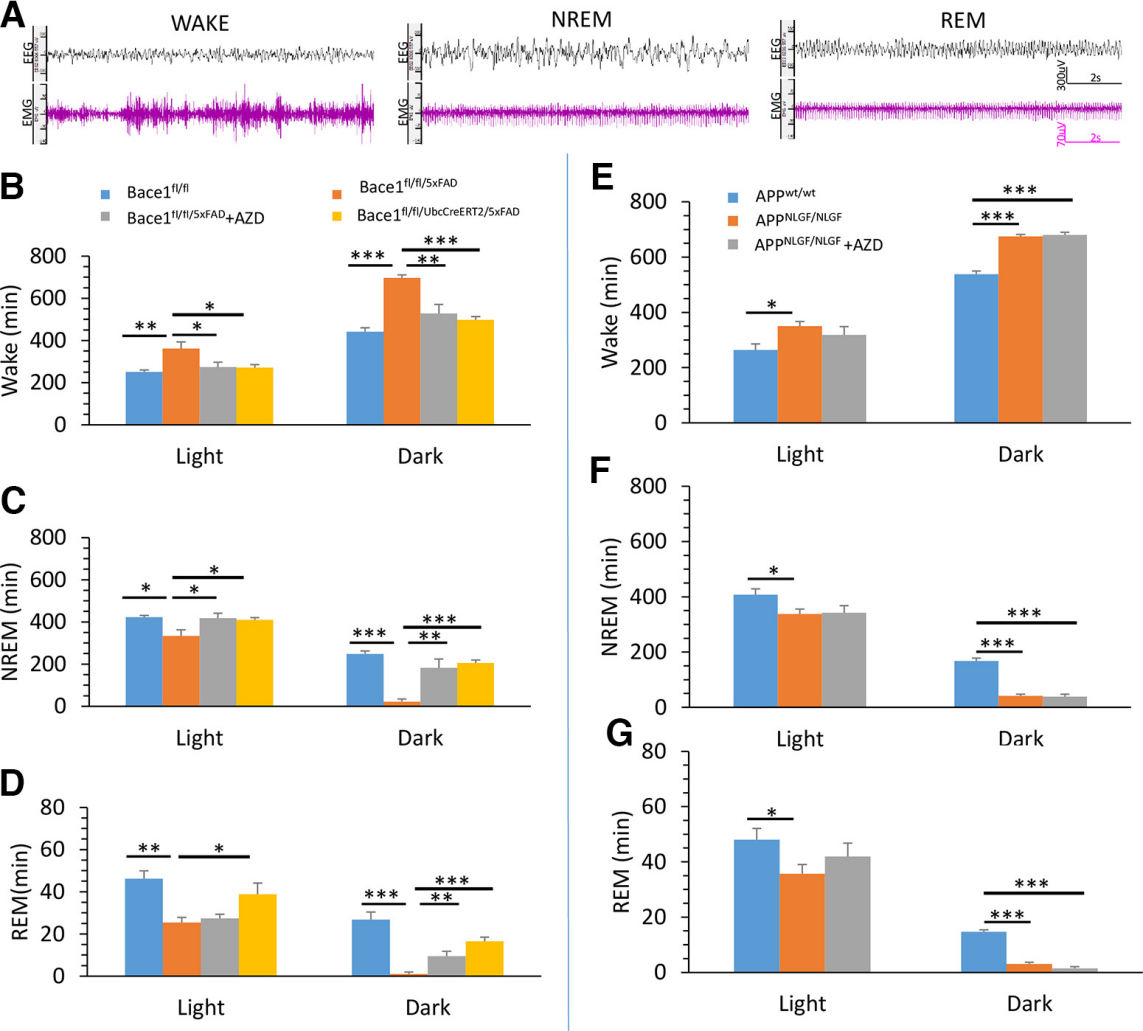
Altered sleep–wake activity was mostly rescued by BACE1 inhibitor in 5xFAD but not in APP KI mice. ***A***, Representative traces of EEG and EMG waveforms in sleep–wake scoring. WAKE is characterized by low-amplitude EEG with mixed high-frequency components combined with high-amplitude EMG. NREM sleep is characterized by relatively high-amplitude EEG with predominant δ and theta components combined with low-EMG tone. REM sleep is characterized by a low-amplitude theta-based EEG combined with muscle atonia and occasional muscle twitches. ***B–G***, Twenty-four hour sleep–wake scoring and analysis on 2 d of video-EEG recordings in 12 h light/12 h dark cycle. Times spent in WAKE (***B***), NREM (***C***), and REM (***D***) are quantified for Bace1^fl/fl^, Bace1^fl/fl/5xFAD^, Bace1^fl/fl/UbcCreER/5xFAD^, and Bace1^fl/fl/5xFAD^ + AZD3293 mice. Times for WAKE (***E***), NREM (***F***), and REM (***G***) for APP^wt/wt^, APP^NLGF/NLGF^, and APP^NLGF/NLGF^ + AZD3293 mice. *p < 0.05; **p < 0.01; *** *p* < 0.001; one-way ANOVA followed by Tukey’s multiple comparisons test. Data are mean ± SEM.

It was previously suggested that amyloid plaques contribute to deficits in sleep in AD (Kang et al., 2009; Musiek and Holtzman, 2016). We sought to study the effect of *Bace1* deletion on these sleep–wake disturbances in 5xFAD and APP KI mice. To this end, we performed sleep–wake scoring and analysis on 2 d of video-EEG/EMG recordings in a 12 h light/12 h dark cycle. Representative EEG and EMG waveforms for WAKE, NREM, and REM sleep are presented in Figure 3*A*.

We found altered WAKE, NREM, and REM times during light and dark phases in 5xFAD mice, while BACE1 inhibitor AZD3293 treatment and genetic *Bace1* deletion in 5xFAD mice had varying effects on restoring these sleep–wake alterations. Time spent in WAKE was significantly increased in Bace1^fl/fl/5xFAD^ mice during the 12 h light phase (361.18 ± 31.36 min in 6 Bace1^fl/fl/5xFAD^ mice vs 250.57 ± 9.81 min in 6 Bace1^fl/fl^ mice, *p* < 0.01), and even more so during the 12 h dark phase (697.33 ± 13.58 min in Bace1^fl/fl/5xFAD^ mice vs 441.47 ± 18.34 min in Bace1^fl/fl^ mice, *p* < 0.001) compared with age-matched Bace1^fl/fl^ controls (Fig. 3*B*). Both AZD3293 treatment and genetic *Bace1* deletion restored the WAKE time back to control levels in both the light and dark phases (273.50 ± 23.29 min in light phase and 527.77 ± 43.11 min in dark phase in Bace1^fl/fl/5xFAD^ mice with AZD treatment; 271.35 ± 13.93 min in light phase and 497.78 ± 14.81 min in dark phase in Bace1^fl/fl/5xFAD/UbcCreER^; Fig. 3*B*).

We also found that time spent in NREM sleep was significantly reduced in Bace1^fl/fl/5xFAD^ mice during the light phase (333.28 ± 29.87 min in Bace1^fl/fl/5xFAD^ mice vs 422.85 ± 7.72 min in Bace1^fl/fl^ mice, *p* < 0.05), and even more significantly during the dark phase (21.67 ± 12.67 min in Bace1^fl/fl/5xFAD^ mice vs 248.28 ± 14.34 min in Bace1^fl/fl^ mice, *p* < 0.001) compared with age-matched controls (Fig. 3*C*). Both AZD3293 treatment and genetic *Bace1* deletion could also restore NREM sleep times back to control levels in both light and dark phases (417.83 ± 23.17 min in light phase and 182.72 ± 40.88 min in dark phase in AZD treatment; 409.73 ± 10.84 min in light phase and 205.73 ± 13.02 min in dark phase in Bace1^fl/fl/UbcCreER/5xFAD^; Fig. 3*C*).

Moreover, time spent in REM sleep was also significantly reduced during both the light phase (25.37 ± 2.44 min in Bace1^fl/fl/5xFAD^ mice vs 46.17 ± 3.74 min in Bace1^fl/fl^ mice, *p* < 0.01) and the dark phase (1.00 ± 1.00 min in Bace1^fl/fl/5xFAD^ mice vs 26.78 ± 3.58 min in Bace1^fl/fl^ mice, *p* < 0.001) compared with age-matched controls (Fig. 3*D*). We found that genetic *Bace1* deletion restored REM sleep in Bace1^fl/fl/5xFAD^ mice in both the light (38.79 ± 5.32 min) and dark (16.45 ± 2.03 min) phases. In contrast, AZD3293 treatment only partially restored REM sleep back to control levels during the dark phase (9.47 ± 2.35 min), but not in the light phase (27.38 ± 1.90 min; Fig. 3*D*).

For APP KI mice, sleep–wake activity was also disrupted: time spent in WAKE for both the light and dark phases was significantly increased compared with APP^wt/wt^ control mice (350.59 ± 16.75 min in APP KI mice vs 264.20 ± 21.84 min in WT mice in light, *n *=* *6, *p* < 0.05, and 675.33 ± 6.41 min in APP KI vs 538.13 ± 11.14 min in WT mice in dark, *p* < 0.001; Fig. 3*E*). Additionally, the time spent in NREM (337.61 ± 18.30 min in APP KI mice vs 407.51 ± 21.56 min in WT mice in light, *p* < 0.05, and 41.58 ± 6.05 min in APP KI vs 167.20 ± 11.05 min in WT mice in dark, *p* < 0.001) and REM (35.72 ± 3.35 min in APP KI mice vs 48.03 ± 4.02 min in WT mice in light, *p* < 0.05, and 3.08 ± 0.54 min in APP KI vs 14.67 ± 0.70 min in WT mice in dark, *p* < 0.001) were significantly reduced in APP KI mice compared with WT controls (Fig. 3*F*,*G*). However, unlike in 5xFAD mice, treatment with the BACE1 inhibitor AZD3293 failed to rescue these sleep–wake disruptions (Fig. 3*E–G*). Overall, these results show increased wakefulness and decreased NREM and REM sleep in both 5xFAD and APP KI mice, but BACE1 inhibitor treatment was only able to rescue these sleep–wake disturbances in 5xFAD mice.

### APP processing is altered in AD mice treated with AZD3293

**Figure 4.**
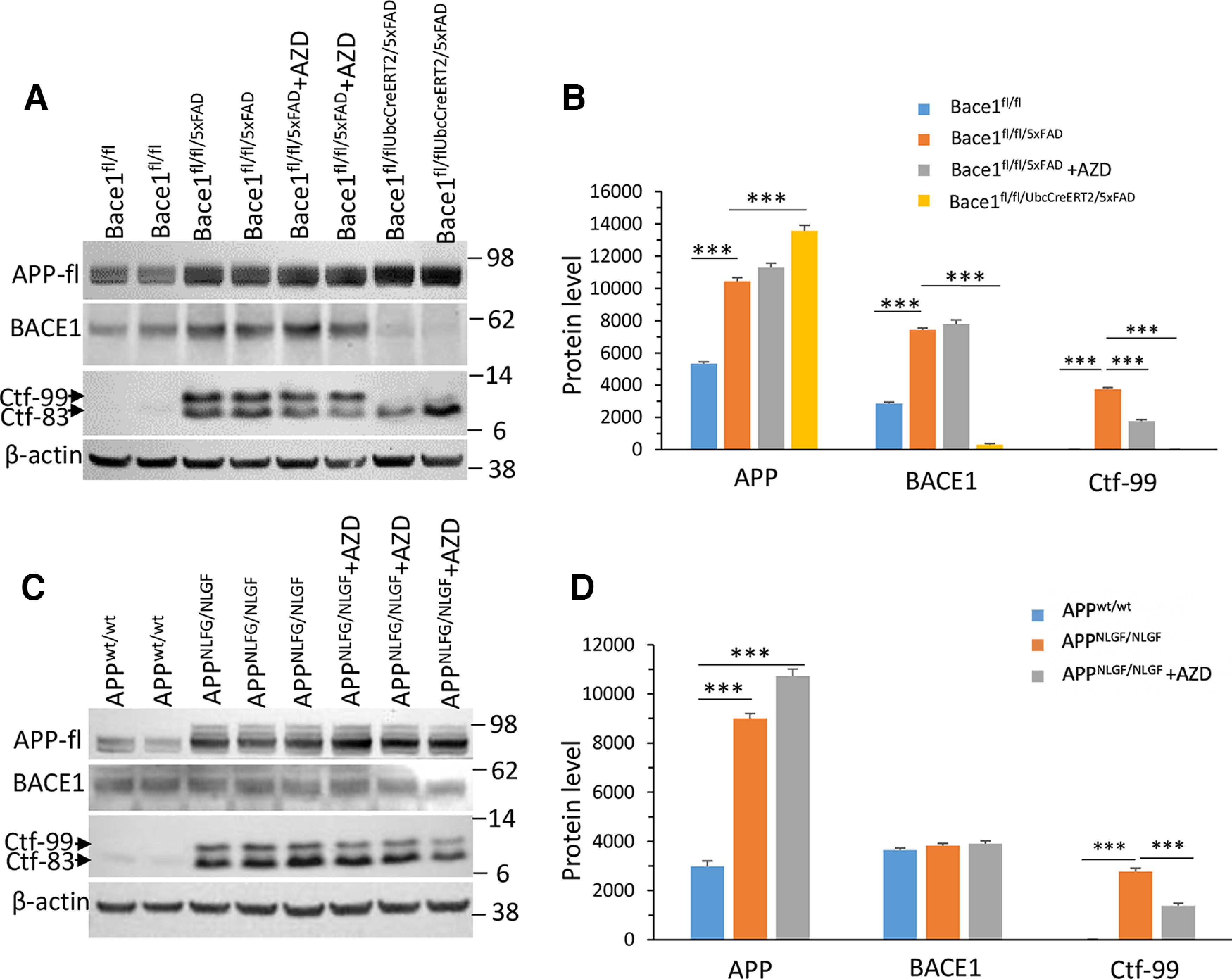
APP processing in AD mice treated with BACE1 inhibitor AZD3293. ***A***, BACE1 protein levels and APP processing products in the cerebral cortex of Bace1^fl/fl^, Bace1^fl/fl/5xFAD^, Bace1^fl/fl/UbcCreER/5xFAD^, and Bace1^fl/fl/5xFAD^ + AZD3293 mice were examined by Western blot. Ctf-99 is a Bace1-cleaved APP C-terminal fragment, which was detected by an APP-C terminal antibody. This antibody also detects Ctf-83, which is a product resulting from α-secretase cleavage of APP. Antibody to β-actin was used to verify equal loading. Blot measurements are in kilodaltons (kDa). ***B***, Bar graphs represent quantification of relative protein levels based on Western blots shown in ***A***. BACE1 protein levels and APP processing products in the cerebral cortex of APP^wt/wt^, APP^NLGF/NLGF^, and APP^NLGF/NLGF^ + AZD3293 mice were examined by Western blot (***C***) and quantified by relative protein level (***D***). Bar graphs are from at least 6 animals in each genotype. *n *=* *3 independent experiments; 2 or 3 animals in each age group were compared side by side. ***p < 0.001 (one-way ANOVA followed by Tukey’s multiple comparisons test). Data are mean ± SEM.

Next, we killed the mice after EEG recordings and performed biochemical analyses on the brains to assess changes in APP processing by Western blot analysis. We found that full-length APP (APP-fl) protein levels were visibly increased in Bace1^fl/fl/5xFAD^ mice compared with Bace1^fl/fl^ controls (*p* < 001, Fig. 4*A*,*B*). AZD3293 treatment in Bace1^fl/fl/5xFAD^ mice did not have a significant effect on levels of APP-fl (*p* = 0.1538, Fig. 4*A*,*B*), while *Bace1*-deleted 5xFAD mice (Bace1^fl/fl/5xFAD/UbcCreER^) exhibited significantly higher levels of APP-fl compared with Bace1^fl/fl/5xFAD^ mice (*p* < 0.001, Fig. 4*A*,*B*). BACE1 protein levels were elevated in Bace1^fl/fl/5xFAD^ mice compared with Bace1^fl/fl^ controls (*p* < 0.001, Fig. 4*A*,*B*). There was no significant difference in BACE1 protein levels in AZD3293-treated versus untreated Bace1^fl/fl/5xFAD^ mice, while *Bace1* genetic deletion (Bace1^fl/fl/5xFAD/UbcCreER^) almost completely abolished BACE1 protein (*p* < 0.001, Fig. 4*A*,*B*). We also measured levels of the BACE1-cleaved APP cleavage product, C99, which was elevated in Bace1^fl/fl/5xFAD^ mice compared with Bace1^fl/fl^ controls (*p* < 0.001, Fig. 4*A*,*B*). C99 levels in AZD3293-treated 5xFAD mice were significantly reduced compared with untreated 5xFAD mice (*p* < 0.001), and C99 were almost undetectable in both Bace1^fl/fl^ and Bace1^fl/fl/UbcCreER/5xFAD^ mice (Fig. 4*A*,*B*).

When we analyzed protein levels in APP KI mice, we found similar profiles from AZD3293 treatment: slightly elevated APP-fl with BACE1 inhibitor treatment with a concomitant reduction in C99 levels; BACE1 levels were unchanged by AZD3293 treatment (Fig. 4*C*,*D*). Together, these data show that ADZ3293 can inhibit BACE1 activity and reduce Aβ generation in both AD mouse models, and the differences in sleep disturbances in 5xFAD mice and APP KI mice following AZD3293 treatment is unlikely because of ADZ3293’s inhibition of APP processing.

### Positive association between epileptiform spikes and plaque load in 5xFAD mice

**Figure 5.**
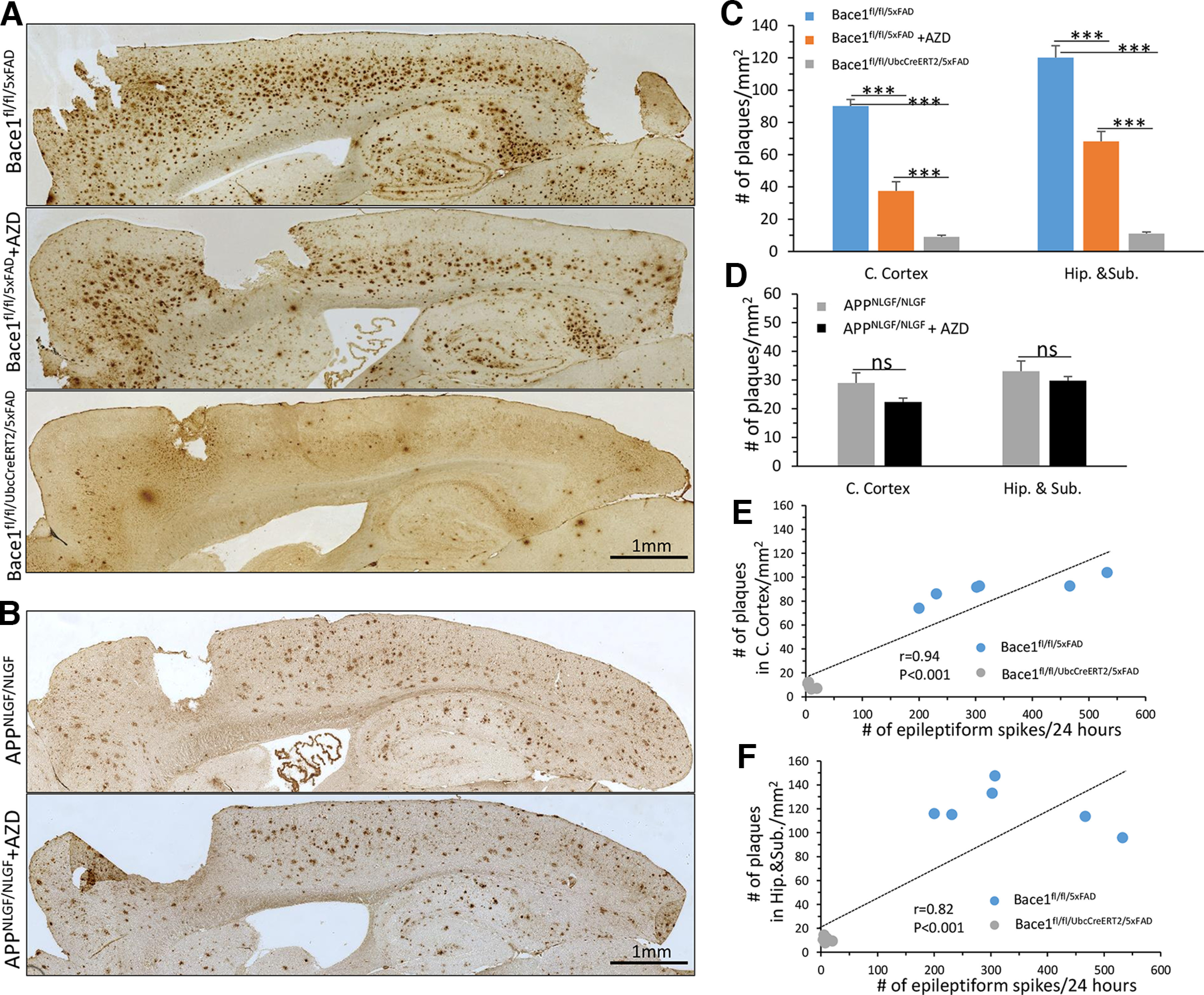
Plaque load was reduced by BACE1 inhibition in 5xFAD but not in APP KI mice. ***A***, ***B***, Representative images of DAB staining of amyloid plaques using 6E10 monoclonal antibody in 10-month-old Bace1^fl/fl/5xFAD^, AZD-treated Bace1^fl/fl/5xFAD^, and Bace1^fl/fl/UbcCreERT2/5xFAD^ mice and APP KI mice treated with or without AZD. Scale bar, 1 mm. ***C***, ***D***, Quantification of plaque load in the cortex and hippocampus and subiculum in 5xFAD and APP KI mice. *n *=* *6 mice per genotype; 10 sections were selected in every 10th per mouse. ***p < 0.001 (unpaired Student’s *t* test). Correlation of plaque load in the cortex (***E***) and hippocampus and subiculum (***F***) plotted against number of epileptiform spikes in 24 h in Bace1^fl/fl/5xFAD^ and Bace1^fl/fl/UbcCreER/5xFAD^ mice. *r* and *p* values are indicated in the plots.

In 5xFAD mice, amyloid plaques first develop at ∼2 months of age, initially in the subiculum and then gradually spreading to other hippocampal and cortical regions (Oakley et al., 2006). To continue our investigation of the underlying changes leading to epileptiform activity and sleep disturbances, we posed the question of whether there was a difference in amyloid plaque loads in these two AD mouse models following *Bace1* deletion or BACE1 inhibition. We quantified amyloid plaques stained by the 6E10 monoclonal antibody, which recognizes the first 16 residues of Aβ. Plaques were manually counted in both cerebral cortex and hippocampus by using ImageJ software (Fig. 5). Consistent with our previous study (Hu et al., 2018), 10-month-old Bace1^fl/fl/5xFAD^ mice exhibited high plaque loads. BACE1 inhibitor treatment led to a significant reduction of plaque numbers by ∼58% in the cerebral cortex (90.21 ± 3.99 plaques per mm^2^ in Bace1^fl/fl/5xFAD^ vs 37.52 ± 5.66 in Bace1^fl/fl/5xFAD^ treated with the BACE1 inhibitor, *p* < 0.001), and by 43% in the hippocampus and subiculum (120.16 ± 7.28 in Bace1^fl/fl/5xFAD^ vs 68.18 ± 6.17 in Bace1^fl/fl/5xFAD^ treated with the BACE1 inhibitor, *p* < 0.001). Age-matched *Bace1*-deleted Bace1^fl/fl/UbcCreER/5xFAD^ mice showed a striking reduction in plaques in both the cerebral cortex and hippocampus, with only a few plaques in the cerebral cortex and hippocampal subiculum (9.02 ± 0.99 in the cortex vs 11.08 ± 0.95 in the hippocampus and subiculum; Fig. 5*A*,*C*). Further, Pearson correlation analysis revealed a strong positive association between epileptiform spikes and plaque load either in the cerebral cortex or hippocampus in Bace1^fl/fl/5xFAD^ and Bace1^fl/fl/UbcCreER/5xFAD^ mice (Fig. 5*E*,*F*; *r* = 0.94, *p* < 0.001 in the cortex and *r* = 0.82, *p* < 0.001 in the hippocampus).

We also quantified plaques in the brains of 10-month-old APP KI mice using the antibody 6E10 (Fig. 5*B*,*D*). As expected, abundant amyloid plaques were found diffusely spread in the cerebral cortex and hippocampus of APP KI mice (Fig. 5*B*). This is consistent with a recent study that also quantified plaque load in APP KI mice, which found that substantial plaque load was evident by 9 months of age, with little further increase through to the oldest ages (Benitez et al., 2021). Surprisingly, we found that no visible or quantifiable reduction in plaque loads in AZD3293-treated APP KI mice in either brain region (Fig. 5*B*,*D*). AZD3293-treated APP KI mice averaged 22.39 ± 1.37 plaques per mm^2^ in the cerebral cortex versus 29.00 ± 3.55 in untreated APP KI mice (*p* = 0.11), and 29.81 ± 1.46 plaques per mm^2^ in the hippocampus of AZD3293-treated APP KI mice versus 33.12 ± 3.51 in untreated (*p* = 0.40). In sum, AZD3293 treatment reduced amyloid plaque number in 5xFAD mice but had minimal effect on plaque number in APP KI mice, although APP processing (i.e., C99 protein level) was inhibited in both AD mouse models. One possibility is that amyloid plaque clearance may be relatively more effective in 5xFAD mice than in APP KI mice.

### Reduced plaque load correlates with improved sleep disturbances in 5xFAD mice

**Figure 6.**
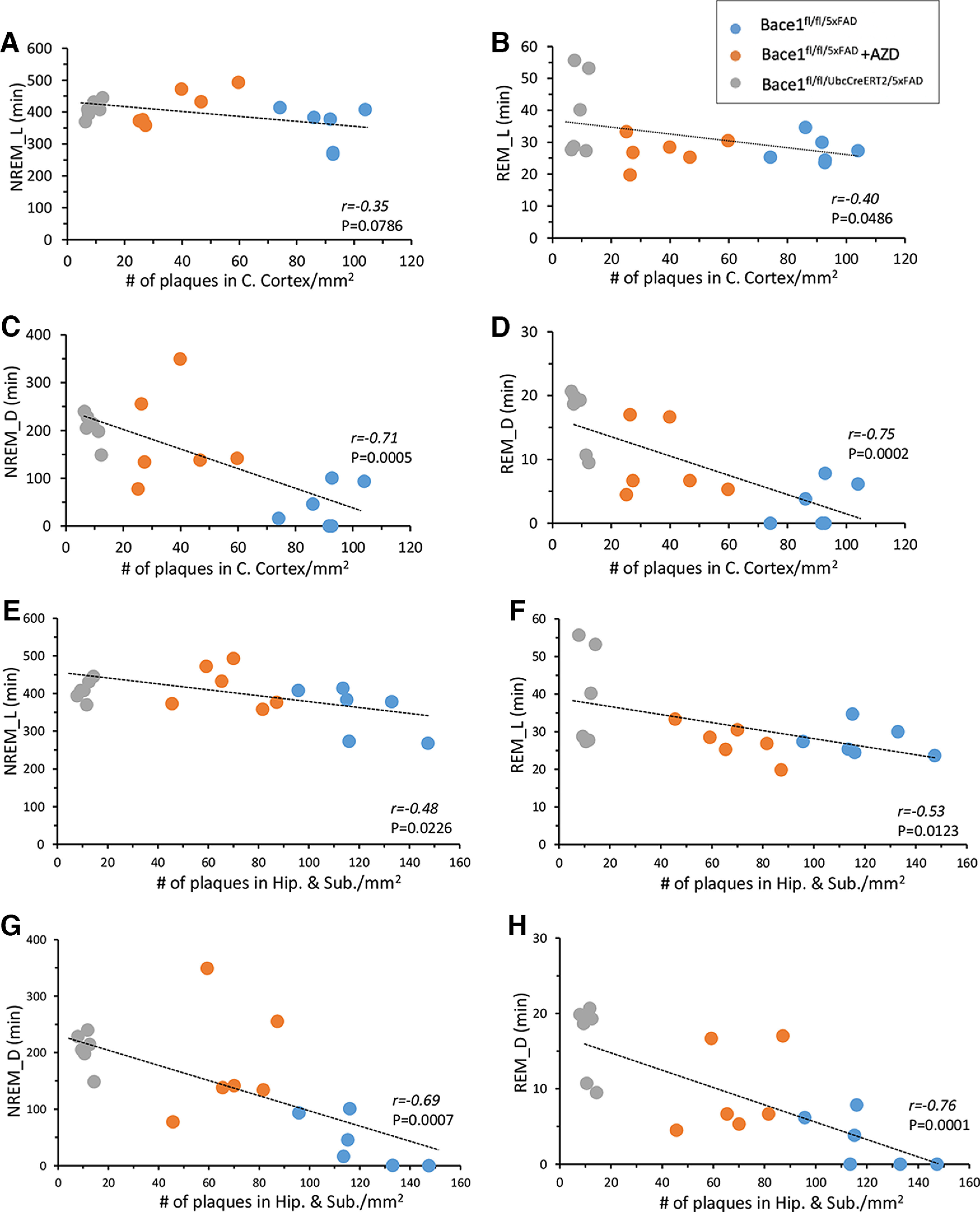
Plaque load in 5xFAD cerebral cortex and hippocampus was negatively correlated with both NREM and REM sleep times. In the cerebral cortex: ***A***, ***B***, Correlation of light-phase NREM and REM time, and (***C***, ***D***) dark-phase NREM and REM time plotted against plaque load in 10-month-old Bace1^fl/fl/5xFAD^, AZD3293-treated Bace1^fl/fl/5xFAD^, and Bace1^fl/fl/UbcCreER/5xFAD^ mice. In the hippocampus and subiculum: ***E***, ***F***, Correlation of light-phase NREM and REM time, and (***G***, ***H***) dark-phase NREM and REM time plotted against plaque load in 10-month-old Bace1^fl/fl/5xFAD^, AZD3293-treated Bace1^fl/fl/5xFAD^, and Bace1^fl/fl/UbcCreER/5xFAD^ mice. *r* and *p* values are indicated in the plots.

To determine whether there is a relationship between plaque load and sleep disturbances seen in 5xFAD mice, we performed correlation analysis of the amyloid plaque counts of untreated Bace1^fl/fl/5xFAD^, AZD3293-treated Bace1^fl/fl/5xFAD^, and Bace1^fl/fl/UbcCreER/5xFAD^ mice and their NREM and REM times during light and dark phase (Fig. 6). Linear regression analysis of these combined cohorts revealed that plaque loads in the cerebral cortex were negatively correlated with both NREM and REM sleep times during both the light phase (Fig. 6*A*,*B*, *r* and *p* values are indicated in the plots) and the dark phase (Fig. 6*C*,*D*, *r* and *p* values are indicated in the plots). A stronger negative association was found during the dark phase (Fig. 6*C*,*D*). Significant negative correlations were also found for plaque loads versus NREM and REM sleep times in the hippocampus during the light phase (Fig. 6*E*,*F*) and dark phase (Fig. 6*G*,*H*). Since the BACE1 inhibitor was unable to reduce plaque counts in either the cerebral cortex and hippocampus of APP KI mice, we did not perform correlation analysis of the plaque counts and sleep time in this AD model. Together, these results demonstrate that sleep impairments correlate with amyloid plaque loads in 5xFAD mice, and both BACE1 inhibition and *Bace1* deletion reduce plaque loads and rescues sleep impairments in this AD mouse model.

### Impaired microglia in APP KI mice have fewer processes and fewer contacts with plaques than 5xFAD mice

In the AD brain, amyloid plaque levels are determined by a balance of plaque deposition and clearance. In addition to other enzymatic and receptor-mediated amyloid clearance (Yoon and Jo, 2012), Aβ aggregates are phagocytosed and degraded by microglia or astrocytes (Rogers et al., 2002; Nielsen et al., 2009). Various studies show that decreased Aβ clearance contributes to the development of AD (Weller et al., 2000). Since AZD3293 significantly decreases plaque load and rescues sleep impairments in 5xFAD but not in APP KI mice (Figs. 3, 5), we asked whether these two AD mouse models would have functional differences in amyloid plaque clearance.

Microglia are the resident immune cells of the brain, and respond to damage signals and neuronal insults by rapidly extending their numerous fine processes to effectively form a 3D seal around sites containing factors released from injured, dead, or dying cells (Hines et al., 2009). Indeed, in AD brains, microglia are clustered around amyloid plaques in both humans (Rozemuller et al., 1986; Mattiace et al., 1990) and mouse models (Bolmont et al., 2008; Meyer-Luehmann et al., 2008), including 5xFAD mice. We recently showed that conditional deletion of *Bace1* in the microglia of adult 5xFAD mice reduced plaque loads, not only through enhanced Aβ uptake but also via more effective clearance of engulfed Aβ via autophagolysosomal degradation machinery (Singh et al., 2022a). To build on this understanding of BACE1’s role in microglial plaque clearance, we therefore investigated whether microglial activation and plaque engulfment in these two AD mouse models are different.

**Figure 7.**
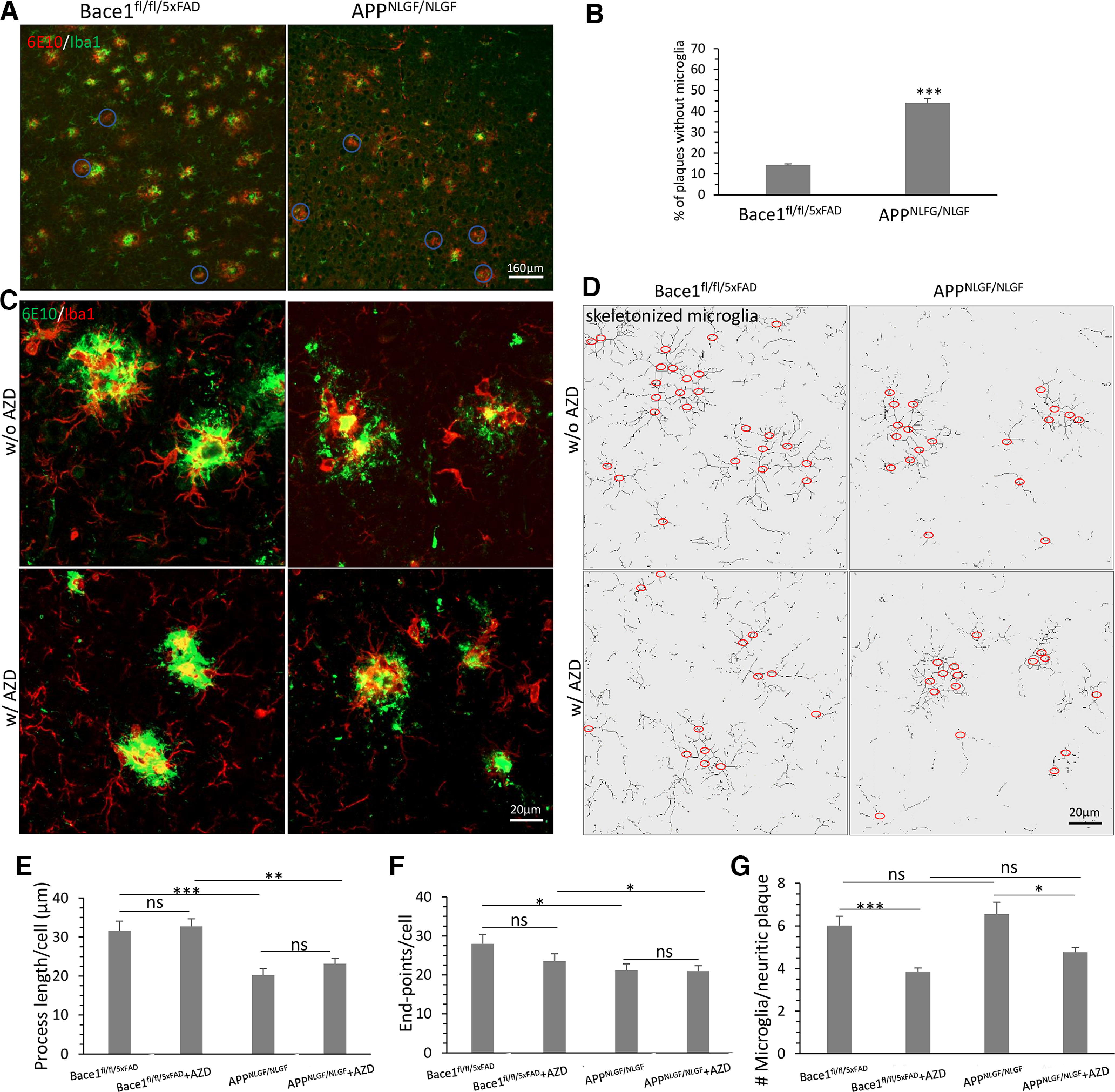
More diffuse plaques and less plaque-associated microgliosis were detected in APP KI mice compared with 5xFAD mice. ***A***, Fluorescent immunolabeling revealed a fewer 6E10-positive plaques (red) not surrounded by activated microglia (green) highlighted with circles in 10-month-old Bace1^fl/fl/5xFAD^ and much more plaques without activated microglia in 10-month-old APP KI mice. Scale bar, 160 μm. ***B***, Quantification of 6E10-positive plaques without activated microglia in the cerebral cortex of Bace1^fl/fl/5xFAD^ and APP KI mice. *n *=* *6 mice per genotype; 10 sections were selected in every 10th per mouse. ***p < 0.001 (unpaired Student’s *t* test). ***C***, Enlarged view of activated microglia (red) and amyloid plaques (green), and (***D***) corresponding skeletonized microglia in both BACE1^fl/fl/5xFAD^ and APP KI mice treated with/without AZD3293. Quantification of average process length (***E***) and endpoints (***F***) of each microglia, and the number of microglia per neuritic plaque (***G***) among the groups. ***G***, Only plaques with microglia were quantified. *n *=* *100 microglia or plaques for each mouse in 6 mice per genotype.

To examine microglial morphology in 5xFAD and APP KI mice, we performed double-staining of brain sections to colabel 6E10-positive amyloid plaques and IBA-1-positive microglia. We found that significantly more microglia were in close contact with amyloid plaques in 5xFAD mice than in APP KI mice (Fig. 7*A*). Morphologically, most 6E10-positive plaques in 5xFAD mice were surrounded by activated microglia. In contrast, only a fraction of 6E10-positive plaques in APP KI mice were surrounded by microglia (Fig. 7*A*). Quantification showed that in APP KI mice, 43.79 ± 2.33% plaques were not surrounded by activated microglia, which is significantly greater than in 5xFAD mice (14.09 ± 0.75% plaques without activated microglia, *p* < 0.001, Fig. 7*B*).

Importantly, we found that the morphology of activated microglia also differed between 5xFAD and APP KI mice. We learn from *in vivo* imaging studies that microglia rely on their processes to continuously survey their microenvironment for signals because of damage, infection, and pathogen-associated molecules (Nimmerjahn et al., 2005), and on encountering damage signals, such as those stimulated by toxic Aβ, undergo the highly conserved microglial response of directed process extension (Davalos et al., 2005; Nimmerjahn et al., 2005; Haynes et al., 2006; Hines et al., 2009; Dissing-Olesen et al., 2014). We found that the activated microglia surrounding plaques in 5xFAD mice had visibly longer and thicker primary processes (Fig. 7*C*, top left), while the activated microglia in APP KI mice had shorter and thinner primary processes (Fig. 7*C*, top right). These observations were also seen in skeletonized microglia traces using the ImageJ software plugin AnalyzeSkeleton (Fig. 7*D*). Morphologic variation in microglial processes may lead to differences in motility and ultimately impacts microglial function (Nimmerjahn et al., 2005; Madry and Attwell, 2015). Thus, the fewer and shorter processes of microglia in APP KI mice suggest weaker microglial phagocytosis of amyloid plaques. Also, AZD3293 treatment did not visibly alter the microglial morphologies in either 5xFAD or APP KI mice compared with their respective untreated controls (Fig. 7*C*,*D*, bottom panels).

Using the skeletonized microglial images (Fig. 7*D*), we quantified these changes in microglial process length, endpoints, and the number of microglia per neuritic plaque (Fig. 7*E–G*). We found that microglia in APP KI mice had shorter processes and fewer process endpoints compared with microglia in 5xFAD mice. This was shown by quantification of process length per cell in Figure 7*E* (31.61 ± 1.34 μm in Bace1^fl/fl/5xFAD^ vs 20.30 ± 1.39 μm in APP KI, *p* < 0.001, *n =*100 cells/mouse in 6 mice each group) and endpoints per cell as shown in Figure 7*F* (27.93 ± 2.45 in Bace1^fl/fl/5xFAD^ vs 21.20 ± 1.61 in APP KI, *p* < 0.05).

Additionally, we found that AZD3293 treatment did not significantly alter microglial morphology in either 5xFAD or APP KI mice compared with their controls (Fig. 7*E*,*F*), but it did reduce microglia number surrounding plaques in both 5xFAD and APP KI mice compared with their respective untreated controls (Fig. 7*G*). There was no significant change in process length per cell in AZD3293-treated Bace1^fl/fl/5xFAD^ (32.75 ± 1.97 μm) compared with untreated 5xFAD (*p* = 0.64, Fig. 7*E*), and AZD3293-treated APP KI (23.16 ± 1.51 μm) compared with untreated (*p* = 0.19, Fig. 7*E*). The number of endpoints per microglia was also not significantly altered in either AZD3293-treated Bace1^fl/fl/5xFAD^ (23.55 ± 1) compared with untreated (*p* = 0.19, Fig. 7*F*), and AZD3293 treated APP KI (21.00 ± 1.40) compared with untreated (*p* = 0.92, Fig. 7*F*). However, the number of microglia per neuritic plaque were significantly reduced in both AD mouse models, as shown in Figure 7*G* (6.01 ± 0.44 in Bace1^fl/fl/5xFAD^ vs 3.84 ± 0.19 in AZD3293-treated Bace1^fl/fl/5xFAD^, *p* < 0.001; 6.56 ± 0.55 in APP KI vs 4.77 ± 0.22 in AZD3293-treated APP KI, *p* < 0.05, *n =*100 plaques/mouse in 6 mice each group).

Another notable observation from our immunostaining of 5xFAD and APP KI brains was the difference in plaque morphology: the plaques in 5xFAD mice were more compact, with well-demarcated margins, compared with more diffuse plaques seen in APP KI mice (Fig. 7*A*,*C*). Recently, a similar phenotype of diffuse plaques and impaired microglial clustering around plaques were found in another APP-overexpressing AD mouse model (Meilandt et al., 2020), which has deficiency in triggering receptor expressed on myeloid cells 2 (*Trem2*), a myeloid-specific AD risk gene that regulates microglial proliferation, phagocytosis, and metabolism (Deczkowska et al., 2020). In our observations, 5xFAD mice had more dense, compact plaques with significant microglial contacts, while APP KI mice had more diffuse plaques with fewer microglial contacts. Our observation suggests that plaque-associated microgliosis is likely altered in APP KI mice. One speculation is that microglial compaction of amyloid into dense plaques may be a protective microglial activity (Meilandt et al., 2020), limiting the exposure of neurons to toxic Aβ. The diffuse plaques and reduced plaque-associated microgliosis in APP KI mice perhaps implies microglial dysfunction as a possible underlying pathology, leading to impaired removal of preformed plaques and the failure to reverse functional deficits like sleep dysfunction in these AD mice.

### BACE1 inhibition differentially alters microglial protein levels in 5xFAD and APP KI mice

**Figure 8.**
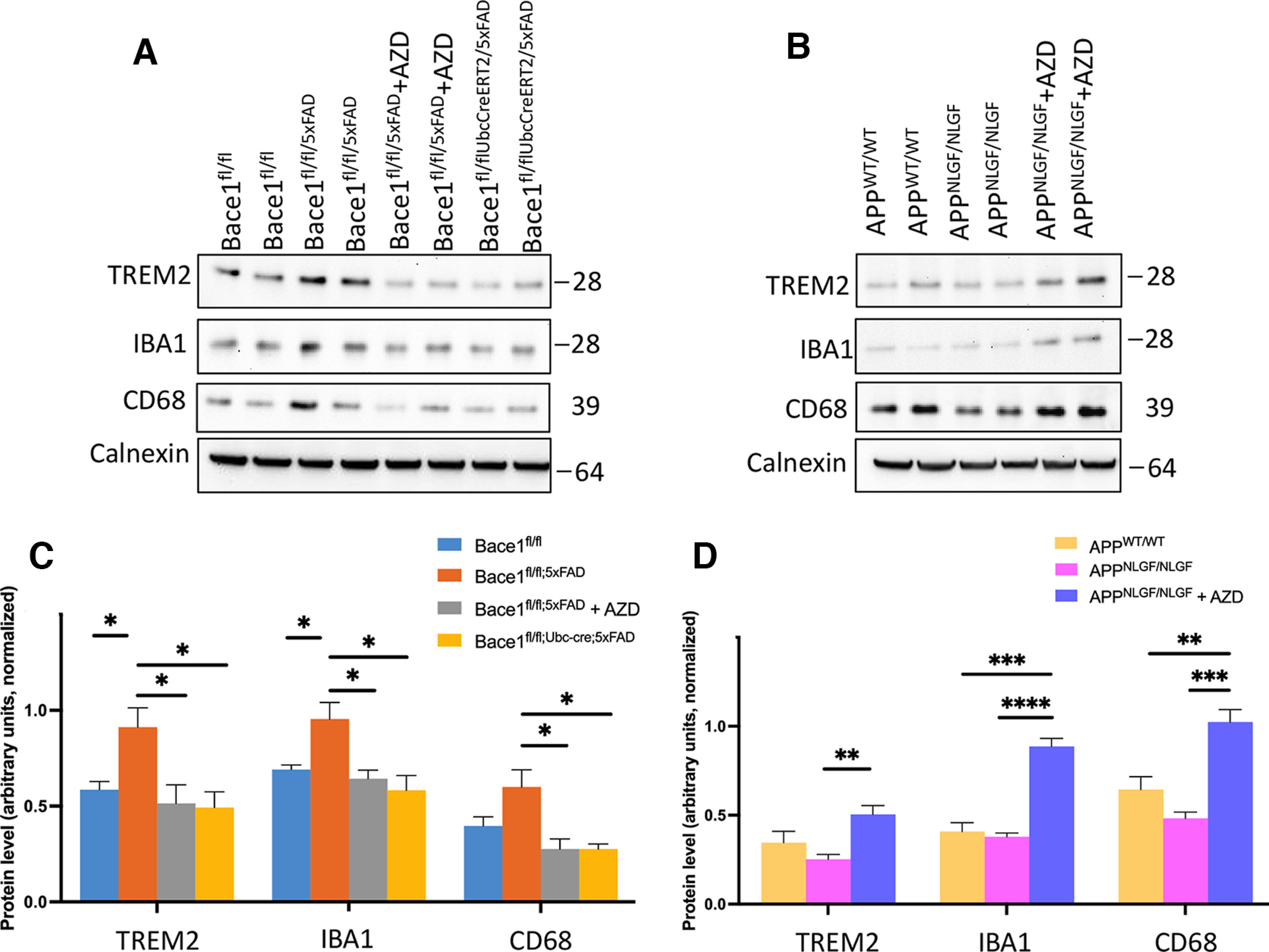
BACE1 inhibition alters microglial activation in AD mouse models. ***A***, ***B***, Immunoblot analysis of TREM2, IBA-1, and CD68 as measured on the cerebral cortex of Bace1^fl/fl^, Bace1^fl/fl/5xFAD^, Bace1^fl/fl/UbcCreER/5xFAD^, Bace1^fl/fl/5xFAD^ + AZD3293, APP^WT/WT^, APP^NLGF/NLGF^, and APP^NLGF/NLGF^ + AZD3293 mice. Antibody to Calnexin was used as loading control. Blot measurements are in kilodaltons (kDa). ***C***, ***D***, Bar graphs represent quantification of relative protein levels based on blots shown in ***A***, ***B***. *n *=* *3 independent experiments; 2 animals in each age group were compared side by side. ***p < 0.001 (one-way ANOVA followed by Tukey’s multiple comparisons test). Data are mean ± SEM.

To further illuminate how *Bace1* deletion or BACE1 inhibition would affect microglial activation and plaque clearance of 5xFAD and APP KI mice, we performed immunoblot assays of microglial-enriched proteins TREM2, IBA-1, and CD68 in the cerebral cortex of each AD mouse model (Fig. 8). We found distinct patterns of altered microglial activation among 5xFAD and APP KI animals: TREM2, IBA-1, and CD68 were significantly increased in Bace1^fl/fl/5xFAD^ animals compared with Bace1^fl/fl^ controls (Fig. 8*A*, quantified in Fig. 8*C*), while these microglial proteins in APP KI mice were trending lower but not significantly changed compared with APP^wt/wt^ controls (Fig. 8*B*, quantified in Fig. 8*D*). We also found opposite patterns of altered microglial activation in 5xFAD and APP KI in response to BACE1 inhibition. Activated microglial proteins were significantly reduced in AZD3293-treated Bace1^fl/fl/5xFAD^ and Bace1^fl/fl/UbcCreER/5xFAD^ mice compared with untreated Bace1^fl/fl/5xFAD^ animals, back to the level of Bace1^fl/fl^ controls (Fig. 8*A*, quantified in Fig. 8*C*). In contrast, microglial proteins, such as TREM2 and IBA-1, in AZD3293-treated APP KI mice were significantly increased relative to untreated APP KI animals, with protein levels exceeding those of APP^wt/wt^ controls (Fig. 8*B*, quantified in Fig. 8*D*), suggesting that AZD3293 treatment has differential effects on APP KI microglial function compared with that of 5xFAD microglia.

## Discussion

Seizures are a debilitating condition with high occurrence in AD and associated with a poor clinical course for individuals with AD (Vöglein et al., 2020). Seizures in AD patients are more common after longer disease duration and advanced neurodegeneration, and also occur more frequently in early-onset patients with genetic mutations associated with excessive brain Aβ accumulation (Joutsa et al., 2017), indicating that synaptic and circuit dysfunction arising from increased Aβ production may be a culprit (Palop and Mucke, 2016). Thus, one rational approach to reduce seizures in AD is inhibition of BACE1, the rate-limiting enzyme for Aβ generation, whose inhibition has been shown to reduce amyloid deposition in both preclinical AD mouse models and clinical studies (Hampel et al., 2021). However, germline *Bace1*-null mice exhibit increased seizures and epileptiform spiking on EEG, and global inhibition of BACE1 alone has failed to improve cognitive functions in patients with AD (Das and Yan, 2019; McDade et al., 2021), indicating a need to refine and optimize this therapeutic strategy. In the present study, we demonstrate that germline *Bace1* deletion specifically in neurons leads to epileptiform activity, while such epileptiform activity is not observed in adult-age *Bace1* deletion or astrocyte-specific deletion. These findings are in line with a report that adult whole-body conditional *Bace1* KOs lack epileptiform abnormalities (Ou-Yang et al., 2018).

Next, we asked the question of whether epileptiform spiking in 5xFAD and APP KI mouse models of AD is alleviated by plaque load/clearance, through *Bace1* adult-age deletion and/or BACE1 inhibition. We found that 5xFAD and APP KI mice exhibit increased epileptiform seizures on EEG, which is in line with previous studies of AD mouse models that show reduced seizure thresholds and spontaneous seizure phenotypes (Moechars et al., 1996; Lalonde et al., 2005; Palop et al., 2007; Westmark et al., 2008; Minkeviciene et al., 2009; Siwek et al., 2015; Reyes-Marin and Nuñez, 2017). Our study of 5xFAD mice found a correlation between the frequency of epileptiform-like discharges and the number of Aβ plaques, similar to a study in APP/PS1 transgenic mice (Reyes-Marin and Nuñez, 2017). Other *ex vivo* and *in vitro* studies suggest that neuronal hyperexcitability and reduced seizure thresholds can be triggered by Aβ itself, even in the absence of amyloid deposits, suggesting a role of cytotoxic soluble forms of Aβ peptide in the generation of aberrant neuronal network activity (Steinbach et al., 1998; Del Vecchio et al., 2004; Minkeviciene et al., 2009). Here, we showed that progressive adult-age *Bace1* deletion in 5xFAD mice reduces amyloid plaque load and EEG spiking, indicating that *Bace1* deletion after early developmental time points will rescue Aβ-mediated abnormal epileptiform activity. These findings suggest that amyloid plaque load in 5xFAD mice may mediate abnormal neuronal and synaptic synchronization, which likely underlies the epileptiform phenotype.

Unexpectedly, BACE1 inhibitor treatment with AZD3293 in 5xFAD and APP KI mice failed to reduce epileptiform activity but rather increased it in the case of APP KI mice. Here, we show, using *ex vivo* extracellular field recordings in the mouse hippocampus, that the burst firing frequency after 4-AP bath application was higher in *Bace1*-null mice treated with AZD3293 compared with untreated *Bace1*-null mice. It appears that AZD3293 enhances synchronous neuronal firing beyond what is seen in global BACE1 inhibition, indicating the dosage of AZD3293 used in this study will cause an off-target effect. Our findings suggest a need to find a safer dose range of AZD3293 for AD treatment.

In addition to seizures, sleep disturbances are also highly comorbid with AD. Sleep plays an important role in the balance of amyloid production and clearance in the brain (Boespflug and Iliff, 2018; Rasmussen et al., 2018; Nedergaard and Goldman, 2020), and early sleep impairments may drive disease pathology and exacerbate disease progression (Musiek et al., 2015, 2018). We show that 5xFAD and APP KI mice both exhibit sleep–wake disturbances, in the form of increased wakefulness and decreased NREM and REM sleep, consistent with sleep architecture changes seen in AD patients (Loewenstein et al., 1982; Prinz et al., 1982; Bliwise et al., 1989; Petit et al., 2004). Our data show a correlation between plaque load and sleep disturbances in 5xFAD mice. Furthermore, both progressive *Bace1* deletion and BACE1 inhibition using AZD3293 rescued sleep–wake disturbances in adult 5xFAD mice. To our knowledge, our study is the first to provide evidence that BACE1 inhibition or deletion reverses sleep deficits in an APP-overexpressing transgenic AD mouse model. Intriguingly, we found that BACE1 inhibitor AZD3293 was not able to rescue similar sleep–wake disturbances in APP KI mice, which may be because of the inability of AZD3293 to clear amyloid plaques in APP KI mice.

Amyloid plaque load in the brain is determined by a balance of plaque deposition and plaque clearance. Aβ clearance is achieved by both enzymatic degradation proteases, such as neprilysin and matrix metalloproteinase-9 (Yoon and Jo, 2012), and nonenzymatic pathways, including interstitial fluid drainage (Weller et al., 2000), active transport across blood vessel walls by clearance receptors, such as low-density lipoprotein receptor-related protein 1 (Deane et al., 2004; Bu et al., 2006; Bell et al., 2007), and uptake by microglial or astrocytic phagocytosis (Rogers et al., 2002; Nielsen et al., 2009). Since microglia are known to play a critical role in amyloid clearance in AD (Heneka et al., 2015; Sarlus and Heneka, 2017), we focused on the potential difference in clearing Aβ by microglia in two different AD mice.

In our study, we found that AZD3293-treated 5xFAD and APP KI mice had differential effects opposite on the level of TREM2 (surface receptor triggering receptor expressed on myeloid cells 2), which is required for Aβ clearance (Gratuze et al., 2018; Griciuc et al., 2019; Zhou et al., 2020; Haure-Mirande et al., 2022). AZD3293 treatment reverted TREM2 to the control levels in 5xFAD mice, while increased TREM2 levels in APP KI mice (Fig. 8*D*). We speculate that this opposite effect is likely because of a recent study on microglial functional impairment in the APP KI mice, and this impairment was not rescued by BACE1 inhibitor AZD3293. APP KI mice have APP clinical mutations in microglia while 5xFAD mice possess the normal APP gene in microglia. We showed that plaque-associated microglia had longer and more abundant primary processes in 5xFAD mice than in APP KI mice (Fig. 7*E*,*F*). These differences are likely microglial function-dependent as suggested in recent studies (Sebastian Monasor et al., 2020; Benitez et al., 2021). Consistently, a recent study showed that this APP KI mouse model is not suitable for investigating Aβ metabolism and clearance because of the Arctic mutation (Sato et al., 2021). Future unbiased RNA sequencing and microglia depletion experiments may provide mechanistic insights for this difference.

In conclusion, our study provides insight into functional impairments of epileptiform activity, sleep disruption, and microglial function in two AD mouse models, and shows that *Bace1* deletion and inhibition rescue deficits in 5xFAD mice, but not APP KI mice. Considering a strong association between plaque load and both epileptiform spiking and sleep impairments in AD mice, our results indicate that safer BACE1 inhibitors will be beneficial for the reducing these disturbances in AD patients.
